# Evidence of widespread sequencing errors in global avian influenza study

**DOI:** 10.1038/s41467-026-78539-0

**Published:** 2026-11-02

**Authors:** Michelle Wille, Ashley C. Banyard, Francesco Bonfante, Andrew C. Breed, Ian H. Brown, Alice Fusaro, Erik A. Karlsson, Nicola S. Lewis, Martha I. Nelson, Leslie D. Sims, David E. Swayne, Marcela M. Uhart, Frank Y. K. Wong

**Affiliations:** 1 Department of Microbiology and Immunology, The University of Melbourne, Peter Doherty Institute for Infection and Immunity, Melbourne, VIC, Australia; 2 Department of Virology, Animal and Plant Health Agency (APHA-Weybridge), Woodham Lane, Addlestone, Surrey UK; 3 WOAH/FAO International Reference Laboratory for Avian Influenza, Newcastle disease virus and Swine Influenza, Animal and Plant Health Agency-Weybridge, Woodham Lane, New Haw, Addlestone, Surrey UK; 4 Division of Comparative Biomedical Sciences, Istituto Zooprofilattico Sperimentale delle Venezie, Legnaro, Padua Italy; 5 School of Veterinary Science, University of Queensland, Brisbane, QLD, Australia; 6 The Pirbright Institute, Pirbright, UK; 7 Virology Unit, WHO H5 Reference Laboratory, Institut Pasteur du Cambodge, Phnom Penh, Cambodia; 8 Worldwide Influenza Centre, WHOCC for influenza reference and research, Francis Crick Institute, London and Royal Veterinary College, University of London, London, UK; 9 Division of Intramural Research, National Library of Medicine, National Institutes of Health, Bethesda, MD USA; 10 Asia Pacific Veterinary Information Services, Montmorency, VIC, Australia; 11 Poultry Diagnostic and Research Center, College of Veterinary Medicine, The University of Georgia, Athens, GA USA; 12 Karen C. Drayer Wildlife Health Center, School of Veterinary Medicine, University of California, Davis, CA, USA; 13 Commonwealth Scientific and Industrial Research Organisation (CSIRO), Australian Centre for Disease Preparedness, Geelong, VIC, Australia

**Keywords:** Influenza virus, Molecular evolution, Microbiology

**arising from** D. L. Wannigama et al.* Nature Communications* https://doi.org/10.1038/s41467-025-59322-z (2025)

Wannigama et al. (2025)^1^ present a study whereby guano samples from infrequently sampled countries were assessed for the presence of avian influenza viruses. Samples were collected from Africa (Madagascar, Mozambique, Somalia), the Middle East (Yemen), South Asia (Maldives, Sri Lanka), South East Asia (Indonesia, Myanmar, the Philippines) and Oceania (Papua New Guinea). The study aims to address critical gaps in global surveillance and provide important data that could enhance understanding of regional viral diversity and evolution, as well as inform high pathogenicity avian influenza (HPAI) incursion risk assessments. However, the data presented and the conclusions drawn from this study are of high concern to the global influenza community. The viral sequence data on which the study is based contradict long-established understanding of global avian influenza evolution, resulting in conclusions that are incongruent with the global evidence base. The publication of erroneous viral sequences in public databases such as GenBank or GISAID hinders rational interpretation of data in a global context, resulting in misleading conclusions that affect disease risk assessments and policy decisions.

A consistently incongruent finding from the study is that the viral hemagglutinin (HA) and neuraminidase (NA) sequences recovered from disparate regions over prolonged time periods share 100% nucleotide identity. This level of genetic conservation is implausible for influenza A viruses, a group of rapidly evolving, negative-sense RNA viruses with a “clock-like” rate of evolution ranging from 1.8 to 8.4 × 10⁻³ nucleotide substitutions per site per year^2,3^, with an average of 2-3 mutations for each replicated genome^4^. These mutation rates lead to continuous genetic variation, even among viruses circulating in the same region or host population, making the detection of 100% identical viruses in wild birds across different continents and years highly unlikely. Detailed analysis of the sequences generated in this study reveals patterns inconsistent with the expected evolutionary trajectories of avian influenza viruses, with a number of examples presented here.

A holistic analysis of the NA sequences of HPAI H5N1 viruses collected in Asia since 1996 reveals the sequences in Wannigama et al. (2025)^1^ to be clear outliers. A root-to –tip regression and scatterplot of residuals obtained from aTempEst^5^ analysis (Fig. 1A) clearly demonstrates that the NA sequences A/feces/Papua_New_Guinea/ID_040/2021 (EPI_ISL_19613364), A/feces/Indonesia/ID_074/2022 (EPI_ISL_19613399), A/feces/Sri_Lanka/ID_170/2021 (EPI_ISL_19613510) are outliers, indicative of improbable evolutionary stasis.

**Fig. 1:**
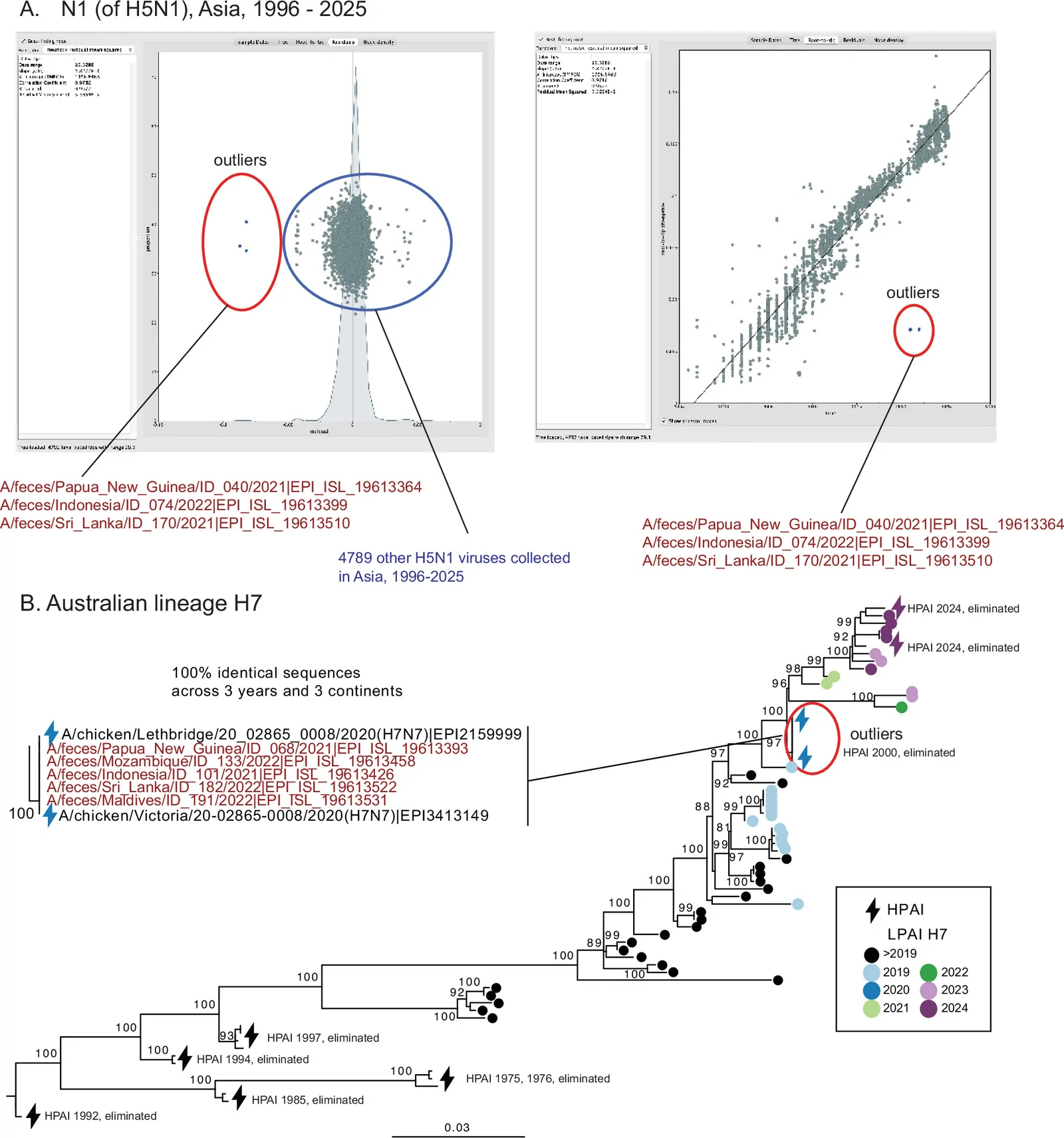
Sequences in this study are inconsistent with expected patterns of evolution and comprise outliers. **A** TempEst residuals and root-to-tip regression analyses for a maximum likelihood phylogeny using 4792 NA sequences of H5N1 viruses collected in Asia between 1996 and 2025 from the GISAID EpiFlu database (accessed 5 July 2025). Sequences generated from Wannigama et al. 2025 are highlighted with a red oval. **B** Maximum likelihood tree of HA sequences in GISAID of Australian lineage H7 viruses. Tips are coloured by year, demonstrating accrued mutations and diversity over time within Australia. Mutation events to HPAI and subsequent eradication indicated by lightning bolt. Five sequences from Wannigama et al. (2025) are 100% similar to an eradicated HPAI H7 strain from 2020. This is in contrast to evolution and genetic diversity in sequences from the same locations in Australia, but across multiple years.

Construction of phylogenetic trees using these data reveals repeated examples of clades comprising numerous sequences from Wannigama et al. (2025) which are 100% identical to those from sequence databases (here GISAID EpiFlu; https://gisaid.org/). For example, Wannigama et al. (2025)^1^ have generated HPAI H7 HA sequences from five countries (Papua New Guinea, Indonesia, Sri Lanka, Maldives and Mozambique) in 2021 or 2022, all of which are 100% identical to HA sequences of an eradicated HPAI H7 virus from Australia (A/chicken/Victoria/20-02865-0008/2020(H7N7)) (EPI3413151) (Fig. 1B). Intensive and subsequence surveillance efforts in Australia confirmed elimination of this virus in 2020 with no spillover into wild birds^6–8^, and therefore it is inconceivable that a virus with 100% similarity would occur on different continents two years later. Similarly, it is implausible that sequences generated in this study from Yemen, the Maldives, Somalia and Papua New Guinea (EPI3724183, see Table S2) fall into a clade of HPAI H5N1 2.3.4.4b circulating in North America, and further, are 100% identical to each other, as welll as sequences from North America (*e.g*. A/Canada goose/New York/NYCVH-23-453/2023(H5N1); EPI3262255). Given that avian influenza viruses have such high rates of evolutionary change, it is high unlikely that these viruses were spread by migrating birds between these locations without accruing a single mutation.

This problem of identical sequences across space and time is widespread in this study. For example, a BLAST search found that all sequences from Papua New Guinea (PNG)(n = 66) had matching HA and NA gene segments with 100% nucleotide identity and length to sequences previously uploaded to GISAID (Table 1, Table S1, S2). Of these, six NA sequences are identical to sequences deposited in GISAID with collection dates spanning 2004-2018 (the samples from Wannigama et al. (2025)^1^ were collected from 2021-2023). Some of these are N1 sequences, which as demonstrated above, are clear outliers in molecular clock analysis. Fourty seven (47) of 66 sequences from PNG are 100% identical to other sequences from Wannigama et al. (2025)^1^, and often from numerous countries and years. In an extreme case, A/feces/Papua New Guinea/ID_043_2023(H5N1) (EPI13724183) is 100% identical to seven other sequences generated in this study (Yemen, Somalia, Maldives). This same sequence is also 100% identical to two sequences from North America (Table S2). Nine sequences from PNG are 100% identical to those from distant flyways which PNG does not fall within [African or North American flyways]. This apparent reservoir of genetic stasis in PNG is inconsistent with the global understanding of avian influenza (Table 1).

**Table 1:** All sequences from PNG (*n* = 66) have 100% nucleotide similarity and length to other sequences in GISAID^a^

Segment	No. sequences from PNG	No. sequences from PNG which have 100% identity and sequence length to sequences in GISAID^b^	No. sequences from PNG which have 100% identity and sequence length to sequences in GISAID^b^ with a sample date prior to 2019^c^ (range of years)	No. sequences from PNG which have 100% identity and sequence length to HPAI lineages which have been eradicated prior to detection in PNG or elsewhere	No. sequences from PNG which have 100% identity and sequence length to sequences in GISAID^b^ which are from “wrong” flyways^d^	No. sequences from PNG which have 100% identity and sequence length to those generated in this study are from “wrong” flyways^d^
HA	32	32	0	1 (H7N7 HPAI)	3	8
NA	34	34	6 (2004-2018)	0	6	6

^a^Blast hits and sequence designations are provided Tables S1, S2.

^b^Uploaded by others; not generated in this study. Blast performed June 2025.

^c^Samples reported in Wannigama et al. (2025) were collected from 2021-2023.

^d^PNG falls into the East Asian Australasian Flyway, which encompasses Australia and parts of Asia. Africa, the middle-east, and USA (here, NY specifically) are not part of this migratory flyway.

Further to issues around apparent evolutionary stasis raised above, is the erroneous assignment of avian H3 viruses to human seasonal-influenza specific clades using NextClade^9^. BLAST analysis of the H3 HA sequences generated confirms that the closest homology is to avian influenza viruses (Table S3). As avian H3 sequence do not have associated genetic clades, clade assignment tools^9^ should have consistently resulted in “unassigned” clades (as demonstrated in Table S3). Rather, the H3 sequences generated in this study were assigned to human-specific clades^1^, including clade 3 C (circulating prior to 2015), 3 C.3a (2014–2016), and 3 C.3a1 (2018–2020), all of which are unrelated to avian viruses^10^^.^

Beyond data issues, no animal ethics or sample collection or movement/export/import permits^11–13^ are listed in this study. Such permits are essential as all countries from which samples have been collected fall under the Convention on International Trade in Endangered Species (CITES) regulations^11^, with six countries also being signatories to the Convention of Biodiversity Nagoya Protocol^12^. Some genomic material and specimens for scientific exchange may be exempt from CITES permits, but require laboratories to be registered and proof of granted exemption is required^11^. Nagoya permits, on the other hand, require Prior Informed Consent (PIC) from the provider country for access to genetic resources, including RNA^12^. Overall, there were no acknowledgements provided that sample collections were exempt from national authorisations from the different countries regardless of their collection in non-protected areas.

There are significant real-world consequences to publishing erroneous data in *Nature Communications* and public repositories. The risk of incorporating and use of these problematic sequences from public repositories is high. Indeed, they have already been included in the Nextstrain avian-flu project (https://nextstrain.org/avian-flu/h5n1/ha/all-time?d=tree,entropy&p=full)(23/07/2025) and are thus already visible to the global community and are hampering genetic assessments. Inclusion of erroneous sequences can interfere with appropriate strain selection for zoonotic candidate vaccine viruses (H5, H7, H9). Australia’s status as free of avian influenza could be called into question if viruses similar to the 2020 outbreak appear to be circulating in wild birds, raising questions about whether the virus was successfully eradicated.

In conclusion, we have identified fundamental issues in the genetic data and resulting conclusions presented by Wannigama et al. (2025)^1^, which require urgent re-evaluation. To maintain the integrity of global influenza surveillance, we further recommend the sequences generated in this study be verified and potentially withdrawn.

## Supplementary information

Supplementary Information
