# Supplementary material for: Evidence of widespread sequencing errors in global avian influenza study
Source: Nat Commun. 2026 Nov 2;17:11335. doi: 10.1038/s41467-026-78539-0 (PMC13728212; doi:10.1038/s41467-026-78539-0)
Supplement: Supplementary file 1 [file 41467_2026_78539_MOESM1_ESM.pdf]

Evidence of widespread sequencing errors in global avian influenza study

Michelle Wille, Ashley Banyard, Francesco Bonfante, Andrew C. Breed, Ian Brown, Alice Fusaro, Erik A. Karlsson, Nicola S. Lewis, Martha I. Nelson, Leslie D. Sims, David E Swayne, Marcela M. Uhart, Frank Y. K. Wong,

**Supplemental Tables**	Page Number
Table S1: Metadata for sequences reported in Table 1	2
Table S2: Detailed list of blast hits for all 66 sequences from PNG	5
Table S3. Top blast hit and NextClade assignment of H3 - HA sequences	18

Table S1. Metadata for sequences from PNG reported in Table 1

**Virus sequence from PNG, generated in this study**<br><br>Accession Designation - Segment	**Sequence(s) in GISAID generated by others from before 2020 which has 100% identity and sequence length**<br><br>Year – Country (Accession)	**Sequence(s) in GISAID generated by others from North America which has 100% identity and sequence length**<br><br>Country (City) - (Accession)	**Sequence(s) in GISAID generated by others from Africa which has 100% identity and sequence length**<br><br>Country - (Accession)	**Sequence(s) generated in this study from Africa/Middle East which has 100% identity and sequence length**<br>Country (Accession)	**HPAI viruses which have been eradicated, or have never been reported east of the Wallace Line**<br><br>Lineage
EPI3724175 A/feces/Papua New Guinea/ID_039/2023(H5N1) - HA				Mozambique (EPI3724352)	Gs/GD 2.3.2.1e
EPI3724177 A/feces/Papua New Guinea/ID_040/2021(H5N1) - NA	2004 **-** Thailand (EPI228373)				
EPI3724176 A/feces/Papua New Guinea/ID_040/2021(H5N1)- NA			Benin (EPI2162090)		
EPI3724179 A/feces/Papua New Guinea/ID_041/2022(H5N1) - HA		USA (New York) (EPI3262231, EPI3257833, EPI3236490, EPI3236474, EPI2500065)			Gs/GD 2.3.4.4b
EPI3724178 A/feces/Papua New Guinea/ID_041/2022(H5N1) - NA		USA (New York) (EPI3262233, EPI3257835, EPI3255560, EPI3236488, EPI3236472)			
EPI3724181 A/feces/Papua New Guinea/ID_042/2021(H5N1) - HA		USA (New York) (EPI3262239, EPI3236466)			Gs/GD 2.3.4.4b
EPI3724180 A/feces/Papua New Guinea/ID_042/2021(H5N1) - NA		USA (New York) (EPI3262241, EPI3255557, EPI3236464)			
EPI3724183 A/feces/Papua New Guinea/ID_043/2023(H5N1) - HA		USA (New York) (EPI3262255, EPI3236482)		Yemen (EPI4208016, EPI4208014, EPI4208012, EPI4208010, EPI3724366)<br>Somalia (EPI3724376)	Gs/GD 2.3.4.4b

EPI3724182 A/feces/Papua New Guinea/ID_043/2023(H5N1) -NA		USA (New York) (EPI3315694, EPI3262257, EPI3236480)		Somalia (EPI3724375)	
EPI3724186 A/feces/Papua New Guinea/ID_045/2023(H4N6) - NA				Madagascar (EPI3724405, EPI3724401)	
EPI3724192 A/feces/Papua New Guinea/ID_048/2022(H7N2) - NA				Madagascar (EPI3724397)	
EPI3724194 A/feces/Papua New Guinea/49/2022(H8N4) - NA	2016 -Australia (EPI2143921)				
EPI3724196 A/feces/Papua New Guinea/50/2022(H8N4) - NA	2018 - Cambodia (EPI1635083)				
EPI3724198 A/feces/Papua New Guinea_51/2022(H8N4) - NA	2016 - Australia (EPI2143921)				
EPI3724200 A/feces/Papua New Guinea/52/2022(H8N4) - NA	2011 - Mongolia (EPI1175868)				
EPI3724206 -A/feces/Papua New Guinea/ID_055/2021(H5N3) - NA	2016 - Australia (EPI2143920)				
EPI3724207 A/feces/Papua New Guinea/ID_055/2021(H5N1) - HA					Gs/GD 2.3.4.4.h
EPI3724208 A/feces/Papua New Guinea/ID_056/2021(H5N3) - NA	2016- Australia (EPI2143920)				
EPI3724209 A/feces/Papua New Guinea/ID_056/2021(H5N1) - HA					Gs/GD 2.3.4.4.h
EPI3724210 A/feces/Papua New Guinea/ID_057/2023(H3N8) - NA				Madagascar (EPI3724405, EPI3724401)	
EPI3724211 A/feces/Papua New Guinea/ID_057/2023(H3N6) - HA				Madagascar (EPI3724408, EPI3724406)	
EPI3724219 A/feces/Papua New Guinea/ID_061/2022(H2N5) - HA				Somalia (EPI3724386)	
EPI3724218 A/feces/Papua New Guinea/ID_061/2022(H2N5) -NA				Somalia (EPI3724385)	
EPI3724223 A/feces/Papua New Guinea/ID_063/2021(H9N2) - HA			Kenya (EPI2043190)		
EPI3724222 A/feces/Papua New Guinea/ID_063/2021(H9N2) - NA			Kenya (EPI2043196)		
EPI3724225 A/feces/Papua New Guinea/ID_064/2022(H5N8) - HA				Somalia (EPI3724388)	Gs/GD 2.3.4.4b

EPI3724224 A/feces/Papua New Guinea/ID_064/2022(H5N8) -NA				Somalia (EPI3724387)	
EPI3724228 A/feces/Papua New Guinea/ID_066/2023(H1N1) - NA		USA (New York) (EPI3262241, EPI3255557, EPI3236464)			
EPI3724231 -A/feces/Papua New Guinea/ID_067/2021(H5N1) - HA					Gs/GD 2.3.4.4.h
EPI3724233 A/feces/Papua New Guinea/ID_068/2021(H7N7) - HA				Mozambique (EPI3724356)	Australian lineage H7N7 HPAI (eradicated in 2020)
EPI3724234 A/feces/Papua New Guinea/ID_069/2021(H6N3) - NA	2016 **-** Australia (EPI2144172)				
EPI3724236 A/feces/Papua New Guinea/ID_070/2023(H10N1) - NA			Benin (EPI2162090)		
EPI3724238 A/feces/Papua New Guinea/ID_071/2023(H7N1) - NA		USA (New York) (EPI3262233, EPI3257835, EPI3255560, EPI3236488, EPI3236472)			

Table S2: Detailed list of blast hits for all 66 sequences from PNG which are 100% identical and have the same sequence length

Accession	Virus	Segment - subtype	Length (bp)	Blast hits in GISAID which are 100% identical and have the same sequence length, generated by others	Other sequences generated in Wannigama et al. (2025) from different countries which are 100% identical and have the same sequence length
EPI3724175	A/feces/Papua New Guinea/ID_039/2023(H5N1)	HA – H5 [HPAI; clade 2.3.2.1.e]	1759bp	EPI3262059 A/chicken/Vientiane/980/2023(H5N1) – 1759bp	EPI3724352 A/feces/Mozambique/ID_130/2022(H5N1) - 1759bp<br>EPI3724307 A/feces/Philippines/ID_105/2022(H5N1) - 1759bp
EPI3724174	A/feces/Papua New Guinea/ID_039/2023(H5N1)	NA – N1	1377bp	EPI3262057 A/chicken/Vientiane/980/2023(H5N1) - 1377bp<br>EPI3262049 A/chicken/Vientiane/001/2022(H5N1) - 1377bp	EPI3724306 A/feces/Philippines/ID_105/2022(H5N1) - 1377bp
EPI3724176	A/feces/Papua New Guinea/ID_040/2021(H5N1)	NA - N1	1371bp	EPI2162090 A/poultry/Benin/21-A-09-031-O/2021(H5N1) -1371bp	EPI3724477 A/feces/Myanmar/ID_002/2022(H5N1) - 1371bp<br>EPI3724425 A/feces/Sri Lanka/ID_170/2021(H5N1) - 1371bp<br>EPI3724244 A/feces/Indonesia/ID_074/2022(H5N1) - 1371bp<br>EPI3724242 A/feces/Indonesia/ID_073/2021(H5N1) - 1371bp
EPI3724177	A/feces/Papua New Guinea/ID_040/2021(H5N1)	NA -N1 Reported as HA, but is NA[1]	1328bp	EPI228373 A/chicken/Nakorn-Patom/Thailand/CU-K2/2004 (H5N1) - 1328bp	EPI3724426 A/feces/Sri Lanka/ID_170/2021(H5N1) - 1328bp<br>EPI3724245 A/feces/Indonesia/ID_074/2022(H5N1) - 1328bp
EPI3724179	A/feces/Papua New Guinea/ID_041/2022(H5N1)	HA – H5 [HPAI; clade 2.3.4.4.b]	1751bp	EPI3262231 A/Canada goose/New York/NYCVH-22-6038/2022(H5N1) - 1751bp<br>EPI3257833 A/red-tailed hawk/New York/NYCVH-22-8477/2022(H5N1) - 1751bp<br>EPI3236490 A/Red tailed hawk/NewYork/8477/2022(H5N1) -1751bp<br>EPI3236474 A/Canada goose/NewYork/NYCVH-22-6038/2022(H5N1) -1751bp<br>EPI2500065 A/Blue Winged teal/MB/FAV-0913/2022(H5N1) -1751bp	EPI3724311 A/feces/Philippines/ID_107/2022(H5N1) - 1751bp<br>EPI3724247 A/feces/Indonesia/ID_075/2023(H5N1) - 1751bp
EPI3724178	A/feces/Papua New Guinea/ID_041/2022(H5N1)	NA – N1	1407bp	EPI3262233 A/Canada goose/New York/NYCVH-22-6038/2022(H5N1) - 1407bp	EPI3724310 A/feces/Philippines/ID_107/2022(H5N1) - 1407bp<br>EPI3724302 A/feces/Indonesia/ID_103/2022(H7N1) - 1407bp

				EPI3257835 A/red-tailed hawk/New York/NYCVH-22-8477/2022(H5N1) - 1407bp<br>EPI3255560 A/red-tailed hawk/NewYork/NYCVH-22-8477/2022(H5N1) -1407bp<br>EPI3236488 A/Red tailed hawk/NewYork/8477/2022(H5N1) -1407bp<br>EPI3236472 A/Canada goose/NewYork/NYCVH-22-6038/2022(H5N1) -1407bp	EPI3724246 A/feces/Indonesia/ID_075/2023(H5N1) - 1407bp
EPI3724181	A/feces/Papua New Guinea/ID_042/2021(H5N1)	HA – H5 [HPAI; clade 2.3.4.4.b]	1751bp	EPI3262239 A/Canada goose/New York/NYCVH-22-9190/2022(H5N1) - 1751bp<br>EPI3236466 A/Canada Goose/NewYork/9190/2022(H5N1) - 1751bp	EPI3724480 A/feces/Myanmar/ID_003/2021(H5N1) - 1751bp<br>EPI3724313 A/feces/Philippines/ID_108/2021(H5N1) - 1751bp<br>EPI3724249 A/feces/Indonesia/ID_076/2021(H5N1) - 1751bp
EPI3724180	A/feces/Papua New Guinea/ID_042/2021(H5N1)	NA – N1	1407bp	EPI3262241 A/Canada goose/New York/NYCVH-22-9190/2022(H5N1) - 1407bp<br>EPI3255557 A/Canada goose/NewYork/NYCVH-22-9190/2022(H5N1) -1407bp<br>EPI3236464 A/Canada Goose/NewYork/9190/2022(H5N1) - 1407bp	EPI3724479 A/feces/Myanmar/ID_003/2021(H5N1)- 1407bp<br>EPI3724312 A/feces/Philippines/ID_108/2021(H5N1)- 1407bp<br>EPI3724292 A/feces/Indonesia/ID_098/2023(H1N1)- 1407bp<br>EPI3724248 A/feces/Indonesia/ID_076/2021(H5N1)- 1407bp
EPI3724183	A/feces/Papua New Guinea/ID_043/2023(H5N1)	HA – H5 [HPAI; clade 2.3.4.4.b]	1751bp	EPI3262255 A/Canada goose/New York/NYCVH-23-453/2023(H5N1) -1751bp<br>EPI3236482 A/Canada goose/NewYork/NYCVH-23-453/2023(H5N1) -1751bp	EPI4208016 >A/feces/Yemen/Socotraarchipelago/ID_10/2022(H5N1) - 1751bp<br>EPI4208014 >A/feces/Yemen/Socotraarchipelago/ID_09/2022/(H5N1) - 1751bp<br>EPI4208012 >A/feces/Yemen/Socotraarchipelago/ID_08/2022(H5N1) - 1751bp<br>EPI4208010 >A/feces/Yemen/Socotraarchipelago/ID_07/2022(H5N1) - 1751bp<br>EPI3724456 A/feces/Maldives/ID_185/2022(H5N1) - 1751bp<br>EPI3724376 A/feces/Somalia/ID_145/2023(H5N1) - 1751bp<br>EPI3724366 A/feces/Yemen/ID_140/2022(H5N1) -1751bp
EPI3724182	A/feces/Papua New Guinea/ID_043/2023(H5N1)	NA – N1	1407bp	EPI3315694 A/Snow_Goose/ON/FAV-1652-2/2022(H5N1) -1407bp	EPI3724455 A/feces/Maldives/ID_185/2022(H5N1) - 1407bp

				EPI3262257 A/Canada goose/New York/NYCVH-23-453/2023(H5N1) -1407bp<br>EPI3236480 A/Canada goose/NewYork/NYCVH-23-453/2023(H5N1) -1407bp	EPI3724375 A/feces/Somalia/ID_145/2023(H5N1) -1407bp<br>EPI3724365 A/feces/Yemen/ID_140/2022(H5N1) -1407bp
EPI3724185	A/feces/Papua New Guinea/ID_044/2022(H4N6)	HA – H4	1695bp	EPI3408458 A/Red-necked stint/Victoria/15159/2020 (H4N8) - 1695bp	n/a
EPI3724184	A/feces/Papua New Guinea/ID_044/2022(H4N6)	NA – N6	1439bp	EPI3115939 A/duck/Bangladesh/17D2026/2022 (H4N6) – 1439bp	n/a
EPI3724187	A/feces/Papua New Guinea/ID_045/2023(H4N6)	HA – H4	1648bp	EPI3101871 A/duck/China/JX01/2023(H4N6) -1648bp<br>EPI3101870 A/duck/China/JX01/2023(H4N6) -1648bp	EPI3724483 A/feces/Myanmar/ID_005/2022(H4N6) -1648bp<br>EPI3724253 A/feces/Indonesia/ID_078/2023(H4N6) -1648bp<br>EPI3724251 A/feces/Indonesia/ID_077/2023(H4N6) -1648bp
EPI3724186	A/feces/Papua New Guinea/ID_045/2023(H4N6)	NA – N6	1360bp	EPI3101867 A/duck/China/JX01/2023(H4N6) -1360bp<br>EPI3101866 A/duck/China/JX01/2023(H4N6) -1360bp	EPI3724405 A/feces/Madagascar/ID_160/2023(H3N6) -1360bp<br>EPI3724401 A/feces/Madagascar/ID_158/2023(H3N6) -1360bp<br>EPI3724336 A/feces/Philippines/ID_120/2023(H3N6) -1360bp<br>EPI3724262 A/feces/Indonesia/ID_083/2023(H3N6) -1360bp<br>EPI3724250 A/feces/Indonesia/ID_077/2023(H4N6) -1360bp<br>EPI3724130 A/feces/Myanmar/ID_017/2023(H3N6) -1360bp
EPI3724189	A/feces/Papua New Guinea/ID_046/2022(H4N6)	HA – H4	1695bp	EPI2386990 A/Wild waterfowl/Australia/02927-0052/2022 – 1695bp	n/a
EPI3724188	A/feces/Papua New Guinea/ID_046/2022(H4N6)	NA – N6	1413bp	EPI2386992 A/Wild waterfowl/Australia/02927-0052/2022 - 1413bp	EPI3724270 A/feces/Indonesia/ID_087/2023(H3N6) -1413bp
EPI3724191	A/feces/Papua New Guinea/ID_047/2022(H4N6)	HA – H4	1695bp	EPI2386990 A/Wild waterfowl/Australia/02927-0052/2022 – 1695bp	n/a
EPI3724190	A/feces/Papua New Guinea/ID_047/2022(H4N6)	NA – N6	1413bp	EPI2386992 A/Wild waterfowl/Australia/02927-0052/2022-1413bp	EPI3724270 A/feces/Indonesia/ID_087/2023(H3N6) -1413bp

EPI3724193	A/feces/Papua New Guinea/ID_048/2022(H2N7)	HA – H2	1748bp	EPI3115957 A/duck/Bangladesh/18D2038/2022(H2N3) -1748bp<br>EPI3115955 A/duck/Bangladesh/18D2038/2022(H2N3) -1748bp	EPI3724259 A/feces/Indonesia/ID_081/2022(H2N7) - 1748bp<br>EPI3724257 A/feces/Indonesia/ID_080/2022(A/H2N7) - 1748bp
EPI3724192	A/feces/Papua New Guinea/ID_048/2022(H7N2)	NA – N7	1436bp	EPI2777046 A/duck/Bangladesh/CEIRS-56485/2023(H1N7) -1436bp<br>EPI2777038 A/duck/Bangladesh/CEIRS-56497/2023(H1N7) -1436bp<br>EPI2777030 A/duck/Bangladesh/CEIRS-56523/2023(H1N7) -1436bp<br>EPI2777014 A/duck/Bangladesh/CEIRS-56577/2023(H1N7) -1436bp<br>EPI2777006 A/duck/Bangladesh/CEIRS-56589/2023(H1N7) -1436bp<br>EPI2776998 A/duck/Bangladesh/CEIRS-56659/2023(H1N7) -1436bp<br>EPI2776990 A/duck/Bangladesh/CEIRS-56665/2023(H1N7) -1436bp<br>EPI2776978 A/duck/Bangladesh/CEIRS-56681/2023(H1N7) -1436bp<br>EPI2776946 A/duck/Bangladesh/CEIRS-56707/2023(H1N7) -1436bp<br>EPI2776933 A/duck/Bangladesh/CEIRS-56711/2023(H1N7) -1436bp<br>EPI2776917 A/duck/Bangladesh/CEIRS-56716/2023(H1N7) -1436bp<br>EPI2776908 A/duck/Bangladesh/CEIRS-56727/2023(H1N7) -1436bp<br>EPI2776901 A/duck/Bangladesh/CEIRS-56731/2023(H1N7) -1436bp<br>EPI2776893 A/duck/Bangladesh/CEIRS-56746/2023(H1N7) -1436bp<br>EPI2776846 A/duck/Bangladesh/CEIRS-56755/2023(H1N7) -1436bp<br>EPI2776839 A/duck/Bangladesh/CEIRS-56791/2023(H1N7) -1436bp	EPI3724397 A/feces/Madagascar/ID_156/2022(H2N7) - 1436bp<br>EPI3724258 A/feces/Indonesia/ID_081/2022(H2N7) - 1436bp<br>EPI3724256 A/feces/Indonesia/ID_080/2022(H2N7) - 1436bp
EPI3724195	A/feces/Papua New Guinea/ID_049/2022(H8N4)	HA – H8	1743bp	EPI3407638 A/duck/Ilan/20WB0032-13/2020 (H8N5) – 1743bp	EPI3724329 A/feces/Philippines/ID_116/2023(H8N4) - 1743bp<br>EPI3724327 A/feces/Philippines/ID_115/2022(H8N4) - 1743bp<br>EPI3724325 A/feces/Philippines/ID_114/2022(H8N4) - 1743bp<br>EPI3724323 A/feces/Philippines/ID_113/2022(H8N4) - 1743bp

					EPI3724321 A/feces/Philippines/ID_112/2022(H8N4) - 1743bp
EPI3724194	A/feces/Papua New Guinea/ID_049/2022	NA – N4	1413bp	EPI2143921 A/wild duck/New South Wales/M16-07034-MD04/2016 – 1413bp	EPI3724320 A/feces/Philippines/ID_112/2022(H8N4) **–** 1413bp
EPI3724197	A/feces/Papua New Guinea/ID_050/2022(H8N4)	HA – H8	1743bp	EPI3407638 A/duck/Ilan/20WB0032-13/2020 (H8N5) – 1743bp	EPI3724329 A/feces/Philippines/ID_116/2023(H8N4) - 1743bp<br>EPI3724327 A/feces/Philippines/ID_115/2022(H8N4) - 1743bp<br>EPI3724325 A/feces/Philippines/ID_114/2022(H8N4) - 1743bp<br>EPI3724323 A/feces/Philippines/ID_113/2022(H8N4) - 1743bp<br>EPI3724321 A/feces/Philippines/ID_112/2022(H8N4) - 1743bp
EPI3724196	A/feces/Papua New Guinea/50/2022(H8N4)	NA – N4	1451bp	EPI1635083 A/duck/Cambodia/C50W8M1/2018 (H7N4) – 1451bp	EPI3724490 A/feces/Myanmar/ID_009/2022(H8N4) – 1451bp
EPI3724199	A/feces/Papua New Guinea/ID_051/2022(H8N4)	HA – H8	1743bp	EPI3407638 A/duck/Ilan/20WB0032-13/2020 (H8N5) – 1743bp	EPI3724329 A/feces/Philippines/ID_116/2023(H8N4) - 1743bp<br>EPI3724327 A/feces/Philippines/ID_115/2022(H8N4) - 1743bp<br>EPI3724325 A/feces/Philippines/ID_114/2022(H8N4) - 1743bp<br>EPI3724323 A/feces/Philippines/ID_113/2022(H8N4) - 1743bp<br>EPI3724321 A/feces/Philippines/ID_112/2022(H8N4) - 1743bp
EPI3724198	A/feces/Papua New Guinea_51/2022(H8N4)	NA – N4	1413bp	EPI2143921 A/wild duck/New South Wales/M16-07034-MD04/2016 – 1413bp	EPI3724320 A/feces/Philippines/ID_112/2022(H8N4) **–** 1413bp
EPI3724201	A/feces/Papua New Guinea/ID_052/2021(H8N4)	HA – H8	1743bp	EPI3407638 A/duck/Ilan/20WB0032-13/2020 (H8N5) – 1743bp	EPI3724329 A/feces/Philippines/ID_116/2023(H8N4) - 1743bp<br>EPI3724327 A/feces/Philippines/ID_115/2022(H8N4) - 1743bp<br>EPI3724325 A/feces/Philippines/ID_114/2022(H8N4) - 1743bp<br>EPI3724323 A/feces/Philippines/ID_113/2022(H8N4) - 1743bp<br>EPI3724321 A/feces/Philippines/ID_112/2022(H8N4) - 1743bp
EPI3724200	A/feces/Papua New Guinea/52/2022(H8N4)	NA – N4	1451bp	EPI1175868 A/duck/Mongolia/565/2011 (A/H8N4) – 1451bp[2]<br>EPI1133986A/duck/Mongolia/258/2011 (A/H8N4) – 1451bp [2]	n/a
EPI3724203	A/feces/Papua New Guinea/ID_053/2023(H8N4)	HA – H8	1743bp	EPI3407638 A/duck/Ilan/20WB0032-13/2020 (H8N5) – 1743bp	EPI3724329 A/feces/Philippines/ID_116/2023(H8N4) - 1743bp

					EPI3724327 A/feces/Philippines/ID_115/2022(H8N4) - 1743bp<br>EPI3724325 A/feces/Philippines/ID_114/2022(H8N4) - 1743bp<br>EPI3724323 A/feces/Philippines/ID_113/2022(H8N4) - 1743bp<br>EPI3724321 A/feces/Philippines/ID_112/2022(H8N4) - 1743bp
EPI3724202	A/feces/Papua New Guinea/ID_053/2023(H8N4)	NA – N4	H8N4 NA	EPI1903126 A/duck/Bangladesh/42987/2020 (A/H8N4) – 1434bp	EPI3724494 A/feces/Myanmar/ID_011/2022(H8N4) **–** 1434bp
EPI3724205	A/feces/Papua New Guinea/ID_054/2023(H8N4)	HA – H8	1743bp	EPI3407638 A/duck/Ilan/20WB0032-13/2020 (H8N5) – 1743bp	EPI3724329 A/feces/Philippines/ID_116/2023(H8N4) - 1743bp<br>EPI3724327 A/feces/Philippines/ID_115/2022(H8N4) - 1743bp<br>EPI3724325 A/feces/Philippines/ID_114/2022(H8N4) - 1743bp<br>EPI3724323 A/feces/Philippines/ID_113/2022(H8N4) - 1743bp<br>EPI3724321 A/feces/Philippines/ID_112/2022(H8N4) - 1743bp
EPI3724204	A/feces/Papua New Guinea/54/2023(H8N4)	NA- N4	1437bp	EPI3354478A/duck/Bangladesh/WF-504/2024(H7N4) -1437bp	EPI3724326 A/feces/Philippines/ID_115/2022(H8N4) – 1437bp
EPI3724207	A/feces/Papua New Guinea/ID_055/2021(H5N1)	HA – H5 [HPAI; clade 2.3.4.4.h]	1732bp	EPI3268579 A/chicken/Vietnam/LBOral74LC/2020 (H5N6) -1732bp	EPI3724497 A/feces/Myanmar/ID_012/2021(H5N3) - 1732bp<br>EPI3724331 A/feces/Philippines/ID_117/2021(H5N3) - 1732bp<br>EPI3724295 A/feces/Indonesia/ID_099/2021(H5N2) - 1732bp<br>EPI3724151 A/feces/Myanmar/ID_027/2022(H5N2) - 1732bp<br>EPI3724149 A/feces/Myanmar/ID_026/2022(H5N2) - 1732bp<br>EPI3724147 A/feces/Myanmar/ID_025/2021(H5N2) - 1732bp
EPI3724206	A/feces/Papua New Guinea/ID_055/2021(H5N3)	NA – N3	1369bp	EPI2143920 A/wild duck/New South Wales/M16-07034-MD04/2016 -1369bp	n/a
EPI3724208	A/feces/Papua New Guinea/ID_056/2021(H5N3)	NA – N3	1369bp	EPI2143920 A/wild duck/New South Wales/M16-07034-MD04/2016 – 1369bp	n/a
EPI3724209	A/feces/Papua New Guinea/ID_056/2021(H5N1)	HA – H5 [HPAI; clade 2.3.4.4.h]	1732bp	EPI3268579 A/chicken/Vietnam/LBOral74LC/2020 (H5N6) -1732bp	EPI3724497 A/feces/Myanmar/ID_012/2021(H5N3) - 1732bp<br>EPI3724331 A/feces/Philippines/ID_117/2021(H5N3) - 1732bp

					EPI3724295 A/feces/Indonesia/ID_099/2021(H5N2) -1732bp<br>EPI3724151 A/feces/Myanmar/ID_027/2022(H5N2) -1732bp<br>EPI3724149 A/feces/Myanmar/ID_026/2022(H5N2) -1732bp<br>EPI3724147 A/feces/Myanmar/ID_025/2021(H5N2) -1732bp
EPI3724211	A/feces/Papua New Guinea/ID_057/2023(H3N6)[3]	HA – H3	1701bp	EPI3426870 A/chicken/China/YC01/2023 (H3N3) -1701bp	EPI3724408 A/feces/Madagascar/ID_161/2022(H3N6) -1701bp<br>EPI3724406 A/feces/Madagascar/ID_160/2023(H3N6) -1701bp<br>EPI3724337 A/feces/Philippines/ID_120/2023(H3N6) -1701bp<br>EPI3724271 A/feces/Indonesia/ID_087/2023(H3N6) -1701bp<br>EPI3724269 A/feces/Indonesia/ID_086/2021(H3N6) -1701bp<br>EPI3724263 A/feces/Indonesia/ID_083/2023(H3N6) -1701bp<br>EPI3724261 A/feces/Indonesia/ID_082/2022(H3N6) -1701bp<br>EPI3724131 A/feces/Myanmar/ID_017/2023(H3N6) -1701bp
EPI3724210	A/feces/Papua New Guinea/ID_057/2023(H3N8)[3]	NA – N6	1360bp	EPI3101867 A/duck/China/JX01/2023(H4N6) -1360bp<br>EPI3101866 A/duck/China/JX01/2023(H4N6) -1360bp	EPI3724405 A/feces/Madagascar/ID_160/2023(H3N6) -1360bp<br>EPI3724401 A/feces/Madagascar/ID_158/2023(H3N6) -1360bp<br>EPI3724336 A/feces/Philippines/ID_120/2023(H3N6) -1360bp<br>EPI3724262 A/feces/Indonesia/ID_083/2023(H3N6) -1360bp<br>EPI3724250 A/feces/Indonesia/ID_077/2023(H4N6) -1360bp<br>EPI3724130 A/feces/Myanmar/ID_017/2023(H3N6) -1360bp
EPI3724213	A/feces/Papua New Guinea/ID_058/2022(H3N6)	HA – H3	1701bp	EPI2777232 A/duck/Mongolia/WKU-42/2022(H3N8) -1701bp<br>EPI2777224 A/duck/Mongolia/WKU-38/2022(H3N8) -1701bp	n/a
EPI3724212	A/feces/Papua New Guinea/ID_058/2022(H3N6)	NA – N6	1413bp	EPI3408352 A/Grey teal/South Australia/22-64233564-49/2022(H4N6) -1413bp<br>EPI3408335 A/Grey teal/South Australia/22-64233564-20/2022(H4N6) -1413bp	EPI3724488 A/feces/Myanmar/ID_008/2022(H4N6) -1413bp<br>EPI3724316 A/feces/Philippines/ID_110/2022(H4N6) -1413bp

				EPI3408281 A/wild waterbird/South Australia/22-68204541-55/2022(H4N6) - 1413bp	
EPI3724215	A/feces/Papua New Guinea/ID_059/2022(H11N9)	HA – N9	1732bp	EPI3219587 A/gull/Shandong/W1805/2022 -1732bp	n/a
EPI3724214	A/feces/Papua New Guinea/ID_059/2022(H11N9)	NA – N9	1433bp	EPI3219589 A/gull/Shandong/W1805/2022 -1433bp	n/a
EPI3724217	A/feces/Papua New Guinea/ID_060/2022(H11N9)	HA-H9	1732bp	EPI3219587 A/gull/Shandong/W1805/2022 -1732bp	n/a
EPI3724216	A/feces/Papua New Guinea/ID_060/2022(H11N9)	NA – N9	1433bp	EPI3219589 A/gull/Shandong/W1805/2022 -1433bp	n/a
EPI3724219	A/feces/Papua New Guinea/ID_061/2022(H2N5)	HA – H2	1737bp	EPI3408070 A/duck/Tainan/20WB0245-94/2020 (H12N5) – 1737bp	EPI3724462 A/feces/Maldives/ID_188/2021(H12N5) - 1737bp<br>EPI3724386 A/feces/Somalia/ID_150/2021(H12N5) - 1737bp<br>EPI3724345 A/feces/Philippines/ID_124/2022(H12N5) - 1737bp<br>EPI3724275 A/feces/Indonesia/ID_089/2022(H12N5) - 1737bp<br>EPI3724135 A/feces/Myanmar/ID_019/2022(H12N5) - 1737bp<br>EPI3724133 A/feces/Myanmar/ID_018/2021(H12N5) - 1737bp
EPI3724218	A/feces/Papua New Guinea/ID_061/2022(H2N5)	NA – N5	1466bp	EPI3408068A/duck/Tainan/20WB0245-94/2020(H12N5) – 1466bp	EPI3724461 A/feces/Maldives/ID_188/2021(H12N5) - 1466bp<br>EPI3724385 A/feces/Somalia/ID_150/2021(H12N5) - 1466bp<br>EPI3724345 A/feces/Philippines/ID_124/2022(H12N5) - 1466bp<br>EPI3724344 A/feces/Philippines/ID_124/2022(H12N5) - 1466bp<br>EPI3724274 A/feces/Indonesia/ID_089/2022(H12N5) - 1466bp<br>EPI3724134 A/feces/Myanmar/ID_019/2022(H12N5) - 1466bp<br>EPI3724132 A/feces/Myanmar/ID_018/2021(H12N5) - 1466bp
EPI3724221	A/feces/Papua New Guinea/ID_062/2023(H?N?)	HA – H9	1717bp	EPI3116000 A/duck/Bangladesh/19D2067/2023(H9N9) -1717bp<br>EPI3115999 A/duck/Bangladesh/19D2067/2023(H9N9) -1717bp	n/a

EPI3724220	A/feces/Papua New Guinea/ID_062/2023	NA – N2	1410bp	EPI2043196 A/chicken/Kenya/440560/2021 (H9N2) -1410bp	n/a
EPI3724223	A/feces/Papua New Guinea/ID_063/2021(H9N2)	HA – H9	1683bp	EPI2043190 A/chicken/Kenya/440560/2021 (H9N2) – 1682bp	n/a
EPI3724222	A/feces/Papua New Guinea/ID_063/2021(H9N2)	NA – N2	1410bp	EPI2043196 A/chicken/Kenya/440560/2021 (H9N2) – 1410bp	n/a
EPI3724225	A/feces/Papua New Guinea/ID_064/2022(H5N8)	HA – H5 [HPAI; clade 2.3.4.4.b]	1762bp	EPI1838501 A/chicken/Miyazaki/E6T/2020(H5N8) - 1762bp<br>EPI1838485 A/chicken/Miyazaki/E1T/2020(H5N8) - 1762bp<br>EPI1838477 A/chicken/Miyazaki/D5T/2020(H5N8) - 1762bp<br>EPI1838469 A/chicken/Miyazaki/D5C/2020(H5N8) - 1762bp<br>EPI1837655 A/chicken/Miyazaki/C8T/2020(H5N8) - 1762bp<br>EPI1837639 A/chicken/Miyazaki/C6T/2020(H5N8) - 1762bp<br>EPI1837631 A/chicken/Miyazaki/C4T/2020(H5N8) - 1762bp	EPI3724444 A/feces/Sri Lanka/ID_179/2023(H5N8) - 1762bp<br>EPI3724388 A/feces/Somalia/ID_151/2021(H5N8) - 1762bp
EPI3724224	A/feces/Papua New Guinea/ID_064/2022(H5N8)	NA – N8	1441bp	EPI2438701 A/environment/Shiga/T23-2/2020(H5N8) - 1441bp<br>EPI2438693 A/environment/Wakayama/T21-5/2020(H5N8) - 1441bp<br>EPI1842191 A/chicken/Miyazaki/I12T/2020(H5N8) - 1441bp<br>EPI1842183 A/chicken/Miyazaki/I11T/2020(H5N8) - 1441bp<br>EPI1839108 A/chicken/Shiga/7T/2020(H5N8) - 1441bp<br>EPI1839084 A/chicken/Shiga/4T/2020(H5N8) - 1441bp	EPI3724443 A/feces/Sri Lanka/ID_179/2023(H5N8) - 1441bp<br>EPI3724387 A/feces/Somalia/ID_151/2021(H5N8) - 1441bp

				EPI1838604<br>A/chicken/Wakayama/4T/2020(H5N8) -<br>1441bp<br>EPI1838596<br>A/chicken/Wakayama/3T/2020(H5N8) -<br>1441bp<br>EPI1838588<br>A/chicken/Wakayama/2T/2020(H5N8) -<br>1441bp<br>EPI1838580<br>A/chicken/Wakayama/1T/2020(H5N8) -<br>1441bp<br>EPI1838508<br>A/chicken/Miyazaki/E9T/2020(H5N8) -<br>1441bp<br>EPI1838500<br>A/chicken/Miyazaki/E6T/2020(H5N8) -<br>1441bp<br>EPI1838492<br>A/chicken/Miyazaki/E3T/2020(H5N8) -<br>1441bp<br>EPI1838484<br>A/chicken/Miyazaki/E1T/2020(H5N8) -<br>1441bp<br>EPI1838476<br>A/chicken/Miyazaki/D5T/2020(H5N8) -<br>1441bp<br>EPI1838468<br>A/chicken/Miyazaki/D5C/2020(H5N8) -<br>1441bp<br>EPI1837654<br>A/chicken/Miyazaki/C8T/2020(H5N8) -<br>1441bp<br>EPI1837646<br>A/chicken/Miyazaki/C7T/2020(H5N8) -<br>1441bp<br>EPI1837638<br>A/chicken/Miyazaki/C6T/2020(H5N8) -<br>1441bp<br>EPI1837630<br>A/chicken/Miyazaki/C4T/2020(H5N8) -<br>1441bp<br>EPI1837574<br>A/chicken/Miyazaki/2T/2020(H5N8) -<br>1441bp	

				EPI1837566 A/chicken/Miyazaki/1T/2020(H5N8) - 1441bp	
EPI3724226	A/feces/Papua New Guinea/ID_065/2021 (H1N1)	HA – H1 (Reported as NA, but is HA)	1774bp	EPI2153455 A/Mallard(Anas platyrhynchos)/South Korea/KNU2021-9/2021(H1N1) – 1774bp (1774)	n/a
EPI3724227	A/feces/Papua New Guinea/ID_065/2021(H1N1)	NA – N1 (Reported as HA, but is NA)	1410bp	EPI2153457A/Mallard(Anas platyrhynchos)/South Korea/KNU2021-9/2021 (H1N1) -1410bp	n/a
EPI3724229	A/feces/Papua New Guinea/ID_066/2023(H1N1)	HA – H1	1752bp	EPI2776984 A/duck/Bangladesh/CEIRS-56681/2023(H1N7) -1752bp<br>EPI2776841 A/duck/Bangladesh/CEIRS-56791/2023(H1N7) -1752bp	EPI3724293 A/feces/Indonesia/ID_098/2023(H1N1) - 1752bp
EPI3724228	A/feces/Papua New Guinea/ID_066/2023(H1N1)	NA – N1	1407bp	EPI3262241 A/Canada goose/New York/NYCVH-22-9190/2022(H5N1) - 1407bp<br>EPI3255557 A/Canada goose/NewYork/NYCVH-22-9190/2022(H5N1) -1407bp<br>EPI3236464 A/Canada Goose/NewYork/9190/2022(H5N1) - 1407bp	EPI3724479 A/feces/Myanmar/ID_003/2021(H5N1) - 1407bp<br>EPI3724312 A/feces/Philippines/ID_108/2021(H5N1) - 1407bp<br>EPI3724292 A/feces/Indonesia/ID_098/2023(H1N1) - 1407bp<br>EPI3724248 A/feces/Indonesia/ID_076/2021(H5N1) - 1407bp
EPI3724231	A/feces/Papua New Guinea/ID_067/2021(H5N1)	HA – H5 [HPAI; clade 2.3.4.4.h]	1732bp	EPI3268579 A/chicken/Vietnam/LBOral74LC/2020 (H5N6) -1732bp	EPI3724497 A/feces/Myanmar/ID_012/2021(H5N3) - 1732bp<br>EPI3724331 A/feces/Philippines/ID_117/2021(H5N3) - 1732bp<br>EPI3724295 A/feces/Indonesia/ID_099/2021(H5N2) - 1732bp<br>EPI3724151 A/feces/Myanmar/ID_027/2022(H5N2) - 1732bp<br>EPI3724149 A/feces/Myanmar/ID_026/2022(H5N2) - 1732bp<br>EPI3724147 A/feces/Myanmar/ID_025/2021(H5N2) - 1732bp
EPI3724230	A/feces/Papua New Guinea/ID_067/2021(H5N2)	NA – N2	1467bp	EPI1974218 A/duck/Vietnam/HN6617/2020 (H3N2) – 1467bp	EPI3724294 A/feces/Indonesia/ID_099/2021(H5N2) - 1467bp<br>EPI3724150 A/feces/Myanmar/ID_027/2022(H5N2) - 1467bp<br>EPI3724148 A/feces/Myanmar/ID_026/2022(H5N2) - 1467bp<br>EPI3724146 A/feces/Myanmar/ID_025/2021(H5N2) - 1467bp
EPI3724233	A/feces/Papua New Guinea/ID_068/2021(H7N7)	HA – H7 [HPAI]	1692bp	EPI3413149 A/chicken/Victoria/20-02865-0008/2020(H7N7) – 1629bp[4]	EPI3724468 A/feces/Maldives/ID_191/2022(H7N7) - 1692bp

				Note: This virus was declared eradicated in 2020[4].	EPI3724450 A/feces/Sri Lanka/ID_182/2022(H7N7) - 1692bp<br>EPI3724356 A/feces/Mozambique/ID_133/2022(H7N7) - 1692bp<br>EPI3724299 A/feces/Indonesia/ID_101/2021(H7N7) - 1692bp
EPI3724232	A/feces/Papua New Guinea/ID_068/2021(H7N7)	NA – N7	1416bp	EPI3413151 A/chicken/Victoria/20-02865-0008/2020(H7N7)- 1416bp	EPI3724467 A/feces/Maldives/ID_191/2022(H7N7) - 1416bp<br>EPI3724449 A/feces/Sri Lanka/ID_182/2022(H7N7)- 1416bp<br>EPI3724298 A/feces/Indonesia/ID_101/2021(H7N7) - 1416bp
EPI3724235	A/feces/Papua New Guinea/ID_069/2021(H6N3)	HA – H6	1744bp	EPI1974795 A/duck/Vietnam/QN6150/2020(H6N6) - 1744bp<br>EPI1974787 A/duck/Vietnam/QN6152/2020(H6N6) - 1744bp<br>EPI1974247 A/Muscovy duck/Vietnam/QN6143/2020(H6N6) - 1744bp<br>EPI1974189 A/Muscovy duck/Vietnam/QN6142/2020(H6N6) - 1744bp	EPI3724301 A/feces/Indonesia/ID_102/2021(H6N3) - 1744bp<br>EPI3724165 A/feces/Myanmar/ID_034/2021(H6N3) - 1744bp<br>EPI3724163 A/feces/Myanmar/ID_033/2021(H6N3) - 1744bp<br>EPI3724161 A/feces/Myanmar/ID_032/2021(H6N3) - 1744bp<br>EPI3724159 A/feces/Myanmar/ID_031/2022(H6N3) - 1744bp<br>EPI3724157 A/feces/Myanmar/ID_030/2021(H6N3) - 1744bp
EPI3724234	A/feces/Papua New Guinea/ID_069/2021(H6N3)	NA – N3	1410bp	EPI2144172 A/ruddy turnstone/King Island/11036/2017 (H3N3) - 1410bp	EPI3724300 A/feces/Indonesia/ID_102/2021(H6N3) - 1410bp
EPI3724237	A/feces/Papua New Guinea/ID_070/2023(H10N1)	HA – H10	1703bp	EPI3358711 A/duck/Bangladesh/17-D-2268/2023(H10N8) – 1703bp	n/a
EPI3724236	A/feces/Papua New Guinea/ID_070/2023(H10N1)	NA – N1	1371bp	EPI2162090 A/poultry/Benin/21-A-09-031-O/2021(H5N1) – 1371bp	EPI3724477 A/feces/Myanmar/ID_002/2022(H5N1) - 1371bp<br>EPI3724425 A/feces/Sri Lanka/ID_170/2021(H5N1) - 1371bp<br>EPI3724244 A/feces/Indonesia/ID_074/2022(H5N1) - 1371bp<br>EPI3724242 A/feces/Indonesia/ID_073/2021(H5N1) - 1371bp
EPI3724239	A/feces/Papua New Guinea/ID_071/2023(H7N1)	HA – H7 [LPAI]	1708bp	EPI3354480 A/duck/Bangladesh/WF-504/2024 (A/H7N4) – 1708bp<br>EPI3354487 A/duck/Bangladesh/WF-506/2024(H7N5) – 1708bp	EPI3724303 A/feces/Indonesia/ID_103/2022 – 1708bp

EPI3724238	A/feces/Papua New Guinea/ID_071/2023(H7N1)	NA – H7	1407bp	EPI3262233 A/Canada goose/New York/NYCVH-22-6038/2022(H5N1) - 1407bp<br>EPI3257835 A/red-tailed hawk/New York/NYCVH-22-8477/2022(H5N1) - 1407bp<br>EPI3255560 A/red-tailed hawk/NewYork/NYCVH-22-8477/2022(H5N1) - 1407bp<br>EPI3236488 A/Red tailed hawk/NewYork/8477/2022(H5N1) - 1407bp<br>EPI3236472 A/Canada goose/NewYork/NYCVH-22-6038/2022(H5N1) - 1407bp	EPI3724310 A/feces/Philippines/ID_107/2022(H5N1) - 1407bp<br>EPI3724302 A/feces/Indonesia/ID_103/2022(H7N1) - 1407bp<br>EPI3724246 A/feces/Indonesia/ID_075/2023(H5N1) - 1407bp

1. There are two NA sequences uploaded for A/feces/Papua New Guinea/ID_041/2022(H5N1). The metadata for EPI3724177 suggests this should be an HA sequence, but it is an NA sequence.
2. Note the 11 year time gap between the detection of A/feces/Papua New Guinea/ID_052/2022(H8N4) and the 100% identical sequences from Mongolia. There is no precedent for evolutionary stasis which would result in no mutations in a virus circulating in nature for 11 years.
3. There are a number of instances wherein the year reported in the designation and metadata are inconsistent. Here, the year reported in the sequence metadata is 2021, whereas the designation is 2023.
4. This virus is from a highly localised HPAI H7N7 outbreak in Victoria, Australia in 2020 This virus was eradicated in 2020, and Australia declared proof of freedom. It was never detected in wild birds. This HPAI H7N7 virus emerged from an Australian lineage LPAI H7 virus. The Australian lineage of H7 is geographically restricted to Australia

Table S3. Top blast hit (GISAID database) and NextClade assignment of H3 - HA sequences

Accession	Virus	Top Blast Hit (not including sequences from this study)	NextClade strain assignment[1]	NextClade strain assignment[2]
EPI3724400	A/feces/Madagascar/ID_157/2023(H3N6)	A/Wild_bird/Mongolia/MN21-2/2021(H3N8) – 100%	unassigned	unassigned
EPI3724402	A/feces/Madagascar/ID_158/2023(H3N6)	A/Wild_bird/Mongolia/MN21-2/2021(H3N8) – 100%	unassigned	unassigned
EPI3724404	A/feces/Madagascar/ID_159/2022(H3N6)	EPI1974219 A/duck/Vietnam/HN6617/2020(H3N2) -100%	failed[3]	unassigned
EPI3724406	A/feces/Madagascar/ID_160/2023(H3N6)	EPI3426870 A/chicken/China/YC01/2023(H3N3) – 100%	unassigned	unassigned
EPI3724408	A/feces/Madagascar/ID_161/2022(H3N6)	EPI3426870 A/chicken/China/YC01/2023(H3N3) – 100%	unassigned	unassigned
EPI3724410	A/feces/Madagascar/ID_162/2021(H3N6)	EPI2593849 A/chicken/Guangdong/5811/2021 (H3N8) -100%	unassigned	unassigned
EPI3724412	A/feces/Madagascar/ID_163/2021(H3N6)	EPI2454652 A/chicken/Henan/S1188/2022(H3N8) – 99%	unassigned	unassigned
EPI3724333	A/feces/Philippines/ID_118/2021(H3N6)	EPI2731199 A/chicken/China/HAUST-16/2023 (H3N3)	unassigned	failed[3]
EPI3724335	A/feces/Philippines/ID_119/2021(H3N6)	EPI1974219 A/duck/Vietnam/HN6617/2020(H3N2) -100%	failed[3]	unassigned
EPI3724337	A/feces/Philippines/ID_120/2023(H3N6)	EPI3426870 A/chicken/China/YC01/2023(H3N3) – 100%	unassigned	unassigned
EPI3724261	A/feces/Indonesia/ID_082/2022(H3N6)	EPI3426870 A/chicken/China/YC01/2023(H3N3) – 100%	unassigned	unassigned
EPI3724263	A/feces/Indonesia/ID_083/2023(H3N6)	EPI3426870 A/chicken/China/YC01/2023(H3N3) – 100%	unassigned	unassigned
EPI3724265	A/feces/Indonesia/ID_084/202(H3N6)	EPI2200697 A/China/ZMD-22-2/2022 (A/H3N8) – 100%	unassigned	failed[3]
EPI3724267	A/feces/Indonesia/ID_085/202(H3N6)	EPI2876733 A/chicken/Anhui/YZU-CKAH02/2022 (A/H3N8) – 100%	unassigned	failed[3]
EPI3724269	A/feces/Indonesia/ID_086/2021(H3N6)	EPI3426870 A/chicken/China/YC01/2023(H3N3) – 100%	unassigned	unassigned
EPI3724271	A/feces/Indonesia/ID_087/2023(H3N6)	EPI3426870 A/chicken/China/YC01/2023(H3N3) – 100%	unassigned	unassigned
EPI3724211	A/feces/Papua New Guinea/ID_057/2023 (H3N6)	EPI3426870 A/chicken/China/YC01/2023(H3N3) – 100%	unassigned	unassigned
EPI3724213	A/feces/Papua New Guinea/ID_058/2022(H3N6)	EPI2777232 A/duck/Mongolia/WKU-42/2022 (A/H3N8) – 100%	unassigned	unassigned

EPI3724123	A/feces/Myanmar/ID_013/2021(H3N6)	EPI2593970 A/chicken/Guangdong/485/2022 (H3N8)	unassigned	failed[3]
EPI3724125	A/feces/Myanmar/ID_014/2022(H3N6)	A/Wild_bird/Mongolia/MN21-2/2021(H3N8) – 100%	unassigned	unassigned
EPI3724127	A/feces/Myanmar/ID_015/2022(H3N6)	A/Wild_bird/Mongolia/MN21-2/2021(H3N8) – 100%	unassigned	unassigned
EPI3724129	A/feces/Myanmar/ID_016/2021(H3N6)	EPI2593849 A/chicken/Guangdong/5811/2021 (H3N8)-100%	unassigned	unassigned
EPI3724131	A/feces/Myanmar/ID_017/2023(H3N6)	EPI3426870 A/chicken/China/YC01/2023(H3N3) – 100%	unassigned	unassigned

1. https://clades.nextstrain.org/, using reference dataset: “Influenza A(H3N2) HA dataset based on reference "A/Wisconsin/67/2005"”
2. https://clades.nextstrain.org/, using reference dataset: “Influenza A(H3N2) HA dataset based on reference "A/Darwin/6/2021"”
3. “Unable to align: seed alignment covers 9.31% of the query sequence, which is less than expected 10.00% (configurable using 'min seed cover' CLI flag or dataset property). This is likely due to low quality of the provided sequence, or due to using incorrect reference sequence.”
